# Home Visiting Interventions and Their Impact on Mental Health, Psychosocial, and Parenting Practice Outcomes of Vulnerable Caregivers: A Systematic Review and Meta-Analysis

**DOI:** 10.3390/ijerph23010063

**Published:** 2025-12-31

**Authors:** Sara Cibralic, Wengtong Wu, Bright Opoku Ahinkorah, Christa Lam-Cassettari, Susan Woolfenden, Jane Kohlhoff, Rebekah Grace, Lynn Kemp, Patrice Johnson, Elisabeth Murphy, April Deering, Shanti Raman, Valsamma Eapen

**Affiliations:** 1 Psychiatry and Mental Health, School of Clinical Medicine, University of New South Wales, Kensington, NSW 2052, Australia; weng_tong.wu@student.unsw.edu.au (W.W.);; 2 Academic Unit of Child Psychiatry (AUCS), South Western Sydney Local Health District & Ingham Institute, Liverpool, NSW 2170, Australia; 3 Community Paediatrics, Sydney Local Health District, Camperdown, NSW 2050, Australia; 4 Central Clinical School, Sydney Medical School, Faculty of Medicine and Health, University of Sydney, Sydney, NSW 2006, Australia; 5 Karitane, Carramar, NSW 2163, Australia; 6 TeEACH Strategic Research Institute, Western Sydney University, Bankstown, NSW 2200, Australia; rebekah.grace@westernsydney.edu.au; 7 School of Nursing and Midwifery, Western Sydney University, Bankstown, NSW 2200, Australia; 8 Health and Social Policy Branch, New South Wales Ministry of Health, St Leonards, NSW 2065, Australia

**Keywords:** home visiting interventions, caregiver outcomes, mental health, psychosocial outcomes, intervention effectiveness, systematic review, meta-analysis

## Abstract

**Highlights:**

**Public health relevance—How does this work relate to a public health issue?**

- Having a caregiver with high vulnerability and complex needs can negatively impact child development, particularly during the early years of life.

**Public health significance—Why is this work of significance to public health?**

- Home visiting interventions can improve caregiver outcomes and, in turn, the caregiving environment.
- Improvements in the caregiving environment can positively impact child health and development.

**Public health implications—What are the key implications or messages for practitioners, policy makers and/or researchers in public health?**

- While home visiting interventions were found to improve a range of caregiver outcomes no one intervention was suitable to address all the varied needs of caregivers with high vulnerability and complex needs.
- Given the limited number of studies on each intervention and the inclusion of primarily female caregivers in participant samples, more research with diverse samples, notably male caregivers, is required.

**Abstract:**

Past reviews have found home visiting interventions to be successful at improving caregiver outcomes. Though, no review has looked specifically at the effect of home visiting interventions on caregivers with high vulnerability and complex needs. This review aimed to examine and synthesis the literature on the impact of home visiting programs administered to caregivers with young children, high vulnerability and complex needs by professionals/paraprofessionals. Interdisciplinary databases, reference lists, and the Home Visiting Evidence of Effectiveness database were searched for articles that fit the inclusion criteria. Together searches resulted in a total of 623 articles, 34 of these articles were included in the final review, all from high-income countries. Twenty-five interventions were implemented across the 34 studies. Findings showed that these interventions were effective at improving a range of mental health, parenting, and family violence outcomes in caregivers with high vulnerability and complex needs. However, weighted mean standardized effect sizes ranged from 0.01–0.24 (small effect), with only one (i.e., practical parenting skills) of the five significantly different from 0 (standardized mean difference 0.24; 95% CI: 0.10, 0.38; *z* = 3.39, *p* = 0.00) and results favoring the control group. Missing information together with considerable variation in interventions, meant that identifying a clear pattern in treatment components that lead to effective verses non-effective interventions was not possible. Further research is therefore needed to assess the effectiveness of these interventions. Trial registration: The University of York Centre for Reviews and Dissemination (PROSPERO) registration number CRD42023460366.

## 1. Introduction

It is well recognised that having a caregiver who experiences high vulnerability and/or has complex needs (i.e., a caregiver with substance abuse difficulties, mentally ill health, family violence, and/or a child protection history) can have negative impacts on a child’s development [1]. This is especially the case during the early years (i.e., 0–5 years), a critical period for brain and neural development, and a time during which caregivers provide the primary care environment for a child [1,2,3,4]. Addressing caregiver mental health, psychosocial needs, and child maltreatment risk during the early years can have positive outcomes on child health and development [5,6]. Home visitation interventions are one approach administered with families who have high vulnerability and/or complex needs and young children to improve both caregiver and child health outcomes [7,8,9].

Home visiting interventions focused on families who are seen to have high vulnerability and/or complex needs are generally offered during the perinatal period, with families able to stay in the program until the child is around two to five years of age [8,9]. The term “home visiting” broadly refers to programs delivered in a home environment and therefore interventions can differ based on their objectives, population of interest, and duration of intervention [10]. There can also be differences in terms of the individuals delivering the program, with home visitors ranging from a single health professionals to teams of professionals, paraprofessionals (i.e., workers who have training in home visiting though are not registered health professionals), and/or unpaid trained laypersons (i.e., volunteer home visitors).

Numerous reviews evaluating the impact of home visiting interventions on families of young children have largely showed positive effects on parental health, attitudes, and behaviours (e.g., [10,11,12,13]). These reviews, however, have not focus exclusively on caregivers who have young children and experience high vulnerability and complex needs. Less is therefore known about what effect home visiting interventions may have on this high-risk population.

## 2. Aim

The aim of the current systematic meta-analytic review was to synthesise and evaluate the literature on caregiver outcomes following the delivery of home visiting interventions to families who have young children and also experience high vulnerability/complex needs. For this review, this included caregivers who experience substance abuse difficulties, mentally ill health, family violence, and/or child protection issues. The review focused on programs delivered by health professionals and/or paraprofessionals. All included studies were synthesised narratively. To get the most precise estimate of the interventions impact, meta-analyses’ were used to calculate pooled effect sizes. Given the considerable variation in home visiting programs and outcomes assessed it was not possible to undertake one meta-analysis, thus independent meta-analyses were undertaken for each outcome.

The aim of this review was to address following research questions:

- What home-visiting interventions have been administered with caregivers who have young children and high vulnerability/complex needs?
- What impacts (primary or secondary) do home-visiting interventions have on caregiver mental health, psychosocial wellbeing, and parenting practices for caregivers with young children and high vulnerability/complex needs?

## 3. Method

This review was registered with the University of York Centre for Reviews and Dissemination (PROSPERO; CRD42023460366). The study protocol was focused on both caregiver and child outcomes, however, given the large volume of literature identified, the authors made the decision to divide the results into two separate reviews- a review on caregiver outcomes and a review on child outcomes [14]. This paper presents the results pertaining to caregiver outcomes.

### 3.1. Search Strategy

The Preferred Reporting Items for Systematic Reviews and Meta-Analysis (PRISMA) guidelines [15] informed the undertaking of both systematic review and meta-analysis. To identify the relevant literature three search strategies were employed: (1) interdisciplinary research databases PsychInfo, Scopus, Embase, PubMed, and CINAHL were searched for studies from database inception up to August 2023 (see Supplementary Table S1 for the exact search terms used in each database); (2) reference lists of studies included in this review were searched manually; (3) the Home Visiting Evidence of Effectiveness (HomVEE) 2023 review and database were searched to identify interventions that were consistent with the inclusion criteria [16].

### 3.2. Inclusion and Exclusion Criteria

Articles were included if they: (1) administered a home visiting intervention; (2) included participants who were pregnant and/or families/caregivers of young children (children aged 0–4 years 11 months or the average study sample age was below 5.5 years); (3) had a sample comprised of caregivers experiencing mentally ill health, substance issue difficulties, family violence, and/or child protection issues; (4) were available in English; and (5) evaluated, using either a standardised measure or qualitative feedback, outcomes related to caregiver mental health, psychosocial needs, and/or parenting practices (i.e., behaviours that parents engage in to care for their child). Of note, while a home visiting intervention had to be administered and a caregiver outcome evaluated, the intervention did need to be specifically designed to target caregiver outcome. That is, the caregiver outcome could have been measured as a secondary outcome.

Articles were excluded if: (1) were not data-based (e.g., books, theoretical papers, reviews); (2) were unpublished dissertations/theses; (3) reported on individual case studies; (4) did not report on caregiver outcomes (i.e., only reported child outcomes); (5) were focused on clinical medical home interventions only; (6) only reported on physical health or birth outcomes; or (7) volunteers administered the home visiting intervention. In cases where it was unclear who delivered the intervention (e.g., the term “home visitor” or “health visitor” was used), intervention manuals and/or websites were searched to determine who generally administered the intervention. Articles were included in the review when it was clear that they were commonly administered by professionals or paraprofessionals. Studies were also excluded if they indicated that the home visiting intervention was delivered by a combination of professionals/paraprofessionals and volunteer home visitors but did not stratify results based on who administered the intervention.

### 3.3. Quality Assessment and Data Analysis

Randomised control trials (RCTs) were evaluated using The Cochrane Collaboration Risk of Bias Assessment Tool [17]. The tool includes seven criteria used to evaluate risk of bias. Each criterion is rated as either low risk (low risk of any bias affecting results), unclear risk (uncertainty about whether the bias will impact results), or high risk (the bias could markedly impact results). Ratings are then used to determine a study’s overall bias risk. Two reviewers individually evaluated each study’s risk of bias then came together to discuss and resolve discrepancies as well as determine the studies’ overall bias risk. Upon discussion, reviewers concluded that achieving participant and professional/paraprofessional blinding was difficult due to the nature of home visiting interventions, thus inadequate randomization and/or high attrition (≥20%) were used to determine whether studies had a high risk of bias.

Non-RCTs were evaluated using the Mixed Methods Appraisal Tool (MMAT; [18]). The MMAT uses five criteria to determine risk of bias. Reviewers indicate “yes,” “no,” or “can’t tell” for each criterion, with “can’t tell” indicating that inadequate detail was provided in the paper to determine the outcome. Two independent reviewers completed quality assessments for all the included studies. Unlike Cochrane Collaboration Risk of Bias Assessment Tool, the MMAT does not support the calculation of an overall quality score. An overall quality score was therefore not calculated, rather the consensus on bias risk was determined through discussions. Please refer to Table 1 for the detailed quality assessments of the included studies.

### 3.4. Data Extraction

The Cochrane Effective Practice and Organization of Care Review Qualitative Evidence Syntheses guidelines were used to guide data extraction [19]. Data was extracted by the second and third authors then checked by the first author. The retrieved information included study components (i.e., design, population, measures, and outcomes) and intervention components (i.e., type, training associated with program delivery, who delivered the intervention, and the problem targeted by the intervention).

### 3.5. Meta-Analysis

Following the Cochrane Collaboration guidelines, when information regarding a variable of interested was available (i.e., sample size, mean, and standard deviation/standard error/confidence intervals) [20] in two or more studies a meta-analysis was undertaken. The Review Manager (RevMan) version 5.4.1 [21] software package was used to undertake the meta-analysis. Continuous variables were calculated using a random effects model with standard mean differences and a 95% confidence interval. Chi-square tests were used to explore heterogeneity of studies. Significance was set at *p* < 0.05, and was quantified using the *I*^2^ statistic, maximum values of 50% identified low heterogeneity [22]. When standard deviation values were not provided, they were calculated using the method outlined by the Cochrane Collaboration [20]. Data is represented visually with forest plots and standardized mean differences are reported with 0.2 indicating a small effect size, 0.5 indicating a median effect size, and 0.8 indicating a large effect size.

**Table 1:** (**a**) The Mixed Methods Appraisal Tool (2018) Results. (**b**) The Cochrane Collaboration Risk of Bias Assessment Tool Results.

**(a)** Non-randomised Studies										
**Citation**										
	**Qualitative Studies**									
	Is the qualitative approach appropriate to answer the research question?	Are the qualitative data collection methods adequate to address the research question?	Are the findings adequately derived from the data?		Is the interpretation of results sufficiently substantiated by data?		Is there coherence between qualitative data sources, collection, analysis and interpretation?			
Zapart et al. (2016) [23]	Yes	Yes	Yes		Yes		Yes			
	**Quantitative nonrandomized**									
	Are the participants representative of the target population?	Are measurements appropriate regarding both the outcome and intervention (or exposure)?	Are there complete outcome data?		Are the confounders accounted for in the design and analysis?		During the study period, is the intervention administered (or exposure occurred) as intended?			
Ammerman et al. (2005) [24]	Yes	Yes	Can’t tell		Can’t tell		Can’t tell			
Ammerman et al. (2011) [25]	Yes	Yes	No		Yes		Yes			
Ammerman et al. (2012) [26]	Yes	Yes	Yes		Yes		Can’t tell			
O’Malley et al. (2021) [8]	Yes	Yes	Yes		No		Yes			
Reuter et al. (2016) [9]	Yes	Yes	Yes		Yes		Yes			
Stacks et al. (2019) [27]	Yes	Yes	No		Can’t tell		Yes			
Stacks et al. (2022) [28]	Can’t tell	Yes	Yes		Yes		Yes			
van Grieken et al. (2019) [29]	Yes	Yes	No		Yes		Can’t tell			
	**Mixed Methods**									
	Is there an adequate rationale for using a mixed methods design to address the research question?	Are the different components of the study effectively integrated to answer the research question?	Are the outputs of the integration of qualitative and quantitative components adequately interpreted?		Are divergences and inconsistencies between quantitative and qualitative results adequately addressed?		Do the different components of the study adhere to the quality criteria of each tradition of the methods involved?			
Giallo et al. (2021) [30]	Yes	Yes	Yes		Yes		Yes			
O’Donnell et al. (2023) [31]	Yes	Yes	Yes		Can’t tell		Yes			
**(b)** Randomised Control Trials										
	**Random Sequence Generation**	**Allocation Concealment**	**Blinding of Participants and Researchers**	**Blinding of Outcome Assessment**		**Incomplete Outcome Data**		**Selective Reporting**	**Anything Else**	**Overall Bias**
Bair-Merritt et al. (2010) [32]	+	+	+	+		+		+	+	Low
Barlow et al. (2007) [33]	+	+	+	+		+		+	+	Low
Bartu et al. (2006) [34]	+	+	?	?		+		+	+	Low
Black et al. (1994) [35]	?	?	?	+		−		+	+	High
Butz et al. (2001) [36]	+	+	?	?		+		+	+	Low
Catherine et al. (2020) [37]	+	−	−	+		+		+	+	Low
Duggan et al. (2004) [38]	+	+	+	+		−		+	+	High
Fergusson et al. (2006) [39]	+	?	?	?		+		+	+	Unclear
Fraser et al. (2000) [40]	+	+	+	+		−		+	+	High
Goldfeld et al. (2019) [41]	+	+	−	+		−		+	+	High
Goldfeld et al. (2021) [42]	+	+	−	+		−		+	+	High
Goldfeld et al. (2022) [7]	+	+	−	+		−		+	+	High
Kemp et al. (2011) [43]	+	+	−	+		−		+	+	High
LeCroy & Lopez (2020) [44]	+	+	?	?		−		+	+	High
Lowell et al. (2011) [45]	+	+	+	?		+		+	+	Low
Mejdoubi et al. (2013) [46]	+	?	?	?		+		+	+	Unclear
Mejdoubi et al. (2014) [46]	+	+	?	+		+		+	+	Low
Oxford et al. (2023) [47]	+	+	?	+		+		+	+	Low
Rosenblum et al. (2020) [48]	+	+	−	+		+		+	+	Low
Sharps et al. (2016) [49]	+	+	+	+		−		+	+	High
Tamaki (2008) [50]	+	+	+	?		+		+	+	Low
Van Doesum et al. (2008) [51]	+	+	?	+		+		+	+	Low
Van Horne et al. (2022) [52]	+	+	+	?		−		+	+	High

Note. All studies met MMAT screening questions criteria S1, “Are there clear research questions?”; and S2, “Do the collected data allow to address the research questions?”. The ‘Can’t tell’ response category means that the paper do not report appropriate information to answer ‘Yes’ or ‘No’. − = Low Risk, + = High Risk, ? = Unclear.

## 4. Results

**Figure 1:**
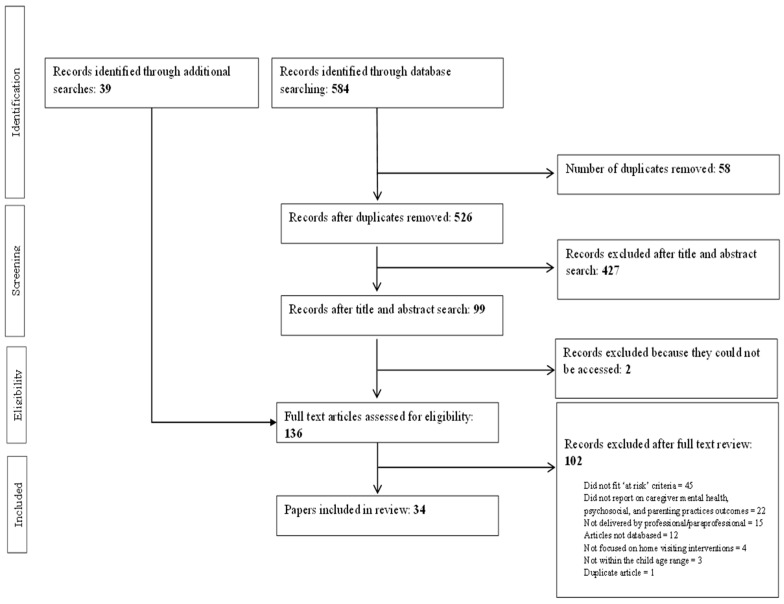
PRISMA flow diagram of included studies.

An overview of the search strategy and number of articles identified at each stage are presented in Figure 1. A total of 584 articles were identified through database searches (0 from CINAHL, 1 from PsychInfo, 5 from Embase, 109 from PubMed, and 469 from Scopus). A total of 526 articles remained after duplicates were removed. Title/abstract screening resulted in the removal of an additional 427 articles. Of the 99 remaining articles, we were unable to locate 2. Additional searches identified a further 39 relevant articles. A total of 136 articles were read during the full text-review stage. This led to the exclusion of a further 102 articles. The remaining 34 articles met inclusion criteria and were included in the present review (see Table 2 for an overview of studies included in the review).

Two reviewers (first author and second or third authors) independently screened all articles, completed full-text reviews, and undertook quality assessments. When disagreements regarding study selection and quality assessment arose they were discussed and resolved. A third reviewer was available in the event that disagreement could not be resolved by the primary reviewers. For title/abstract screening inter-rater agreement was 88.1% while for and full-text screening it was 66.1%.

**Table 2:** Overview of included studies.

Article	Target Problem	Intervention	Study Design	Country	Demographics
Ammerman et al. (2005) [24]	Mental health (depression)	IH-CBT *	Quasi-experimental	USA	N = 26 women, M age = 22.52 (SD 3.95).
Ammerman et al. (2011) [25]	Mental health (depression)	IH-CBT *	Quasi-experimental	USA	N = 307 women; Intervention n = 64, M age = 22.57 (SD 4.96); Control n = 241, M age = 20.15 (SD 4.18).
Ammerman et al. (2012) [26]	Mental health (depression)	IH-CBT *	Quasi-experimental	USA	N = 60 women; M age = 22.4 years (SD 5.0); Child M age = 152.0 days (SD 73.0).
Bair-Merritt et al. (2010) [32]	Partner violence	Hawaii Healthy Start Program	RCT	USA	N = 643 women; Intervention n = 373, M age not provided; Control n = 270, M age not provided.
Barlow et al. (2007) [33]	Mental health (depression)	Health visiting intervention	RCT	United Kingdom	N = 131 women; Intervention n = 68, M age not provided; Control n = 63, M age not provided.
Bartu et al. (2006) [34]	Mental health (substance use)	Home visiting intervention	RCT	Australia	N = 152 women; Intervention n = 76 women, median age = 27 years (17–39), Control group n = 76 women, median age = 25 years (18–41).
Black et al. (1994) [35]	Mental health (substance use)	SPICE	RCT	USA	N = 60 women; Intervention n = 31, M age = 26.4 (SD 0.9); Control n = 29, M age = 27.9 (SD 0.7)
Butz et al. (2001) [36]	Mental health (substance use)	Home visiting intervention	RCT	USA	N = 117 women; Intervention n = 59, M age = 28.0 (SD 4.6); Control n = 58, M age = 28.9 (SD 4.5)
Catherine et al. (2020) [37]	Mental health (substance use)	Nurse-Family Partnership	RCT	Canada	N = 739 women; Intervention n = 368, M age not provided; Control n = 371, M age not provided.
Duggan et al. (2004) [38]	Child maltreatment	Hawaii Healthy Start Program	RCT	USA	N = 643 women; Intervention n = 373, M age = 23.7 years (SD 5.8); Control n = 270, M age = 23.3 years (SD 5.8)
Fergusson et al. (2005) [39]	At risk families (including mental health difficulties and domestic violence)	Early start	RCT	New Zealand	N = 391 women; Intervention n = 184 women, M age not provided; Control n = 207, M age not provided.
Fraser et al. (2000) [40]	Child maltreatment	Home visiting intervention	RCT	Australia	N = 181 women; Intervention n = 90 women, M age = 25.72 years (SD 5.61); Control n = 91 women, M age = 26.67 years (SD 6.08).
Giallo et al. (2021) [30]	Child maltreatment	HoPES	Mixed methods	Australia	N = 30 families; mother n = 29, M age = 28.9 years (SD 7.1); father n = 11, M age = 34.09 years (SD 10.8); children n = 31, M age = 14.4 months (SD 9.7), 46.7% female.
Goldfeld et al. (2019) [41]	Women experiencing adversity (including mental health difficulties)	right@home	RCT	Australia	N = 722 women; Intervention n = 306 women, M age = 27.6 years (SD 6.1); Control n = 359 women, M age = 27.4 years (SD 6.2).
Goldfeld et al. (2021) [42]	At risk women (including mental health difficulties)	right@home	RCT	Australia	N = 495 women; Intervention n = 255 women, M age = 27.6 years (SD 5.9); Control n = 240 women, M age = 28.3 years (SD 6.4).
Goldfeld et al. (2022) [7]	Women experiencing adversity (including mental health difficulties)	right@home	RCT	Australia	N = 426 women; Intervention n = 225 women, M age = 27.9 years (SD 6.0); Control n = 201 women, M age = 28.7 years (SD 6.4).
Kemp et al. (2011) [43]	Women experiencing adversity (including mental health difficulties and domestic violence)	Sustained structured nurse home visiting program	RCT	Australia	N = 208 women; Intervention n = 111 women, M age = 27.6 years (SD 6.7); Control n = 97 women, M age = 27.7 years (SD 5.9).
LeCroy & Lopez (2020) [44]	Mental health	Healthy Families Arizona	RCT	USA	N = 245 families; Intervention n = 98 women, M age not provided; Control n = 147 women, M age not provided. Father demographics not provided.
Lowell et al. (2011) [45]	Child emotional/behavioural problems and/or parent psychosocial risk	Child FIRST	RCT	USA	N = 157; Intervention n = 78 mothers, mother M age = 27.7 years (SD = 7.0), child M = 19.0 months, (SD = 9.2), 42.3% male; Control n = 79, mother M = 26.9 years, (SD = 6.9), child M = 18.0, (SD = 8.8), 45.6% male.
Mejdoubi et al. (2013) [46]	Partner violence	VoorZorg	RCT	Netherlands	N = 460 women; Intervention n = 237 women, M age = 19.5 years (SD 2.8); Control n = 223 women, M age = 19.2 years (SD 2.6)
Mejdoubi et al. (2014) [53]	At risk women (including substance use and domestic violence)	VoorZorg	RCT	Netherlands	N = 460 women; Intervention n = 223, M age = 19.5 years (SD 2.8); Control n = 237; M age = 19.2 years (SD 2.6).
O’Malley et al. (2021) [8]	Mental health (substance use)	TIES	Quasi-experimental	USA	N = 220 women, M age not provided.
O’Donnell (2023) [31]	Families with multiple risk factors (e.g., family violence, substance use, mental health concerns, Child Protection involvement)	Cradle to Kinder	Mixed methods	Australia	Quantitative component: N = 57 families, metropolitan families n = 24 (29% = Aboriginal or Torres Strait Islander), mother M age = 19.33 years (SD = 2.31), father involvement in program = 33%; rural families n = 33 (32% = Aboriginal or Torres Strait Islander), mother M = 18.44 (SD = 0.68, father involvement in program = 48%. Qualitative component: N = 14, 11 months, 3 fathers, M age = 22.5 (SD = 3.04), 29% = Aboriginal or Torres Strait Islander.
Oxford et al. (2023) [47]	Mental health	Moms and Babies Program (Promoting First Relationships)	RCT	USA	N = 252 women; Intervention n = 127, [low distress: n = 85, mother M age = 28.81 years (SD 5.67), child’s M age = 1.85 months (SD 0.46); high distress n = 42, mother M age = 28.64 years (SD 6.25), child’s M age = 1.83 months (SD 0.45); Control n = 125, [low distress: maternal M age = 27.94 years (SD 5.27), child M age = 1.82 months (SD 0.45); high distress: maternal M age = 26.26 years (SD 6.22), child M age = 1.92 months (SD 0.53)].
Reuter et al. (2016) [9]	Mental Health	PFF	Quasi-experimental	USA	N = 215 caregivers, M age = 34.04 years (SD 9.65). Child protective services -referred families n = 84, M age = 37.85 years (SD 10.07); Prenatal-referred families n = 131, M age = 31.49 (SD 8.47).
Rosenblum et al. (2020) [48]	Parents with adverse childhood experiences	Michigan Model of IMH-HV	RCT	USA	N = 62 families; Intervention n = 32, mother M age = 32.38 years (SD 5.72), child M age = 23.59 months (SD 6.57).
Sharps et al. (2016) [49]	Partner violence	DOVE ^a^	RCT	USA	N = 239 women, Intervention n = 124, M age = 24.3 (SD 5.6); Control n = 115, M age = 23.4 (SD 5.4).
Stacks et al. (2019) [27]	Child maltreatment	Michigan Model of IMH-HV	Quasi-experimental	USA	N = 16 parents, 68% female, M age = 21.69 years (SD 4.53), M child age 18.57 months (SD 7.1).
Stacks et al. (2022) [28]	Mental health	Michigan Model of IMH-HV	Quasi-experimental	USA	N = 75 women, M age = 26.67 years (SD 6.11); child M age = 9.64 months.
Tamaki (2008) [50]	Mental health (depression)	Home visiting intervention	RCT	Japan	N = 16 women; Intervention n = 7, M age = 33.86 years (SD 3.02); Control n = 9, M age = 33.78 (SD 5.33).
van Doesum et al. (2008) [51]	Mental health (depression)	Home visiting intervention	RCT	Netherlands	N = 85 women; Intervention n = 36 women, M age = 30.4 years (SD 4.1); Control n = 35 women, M age = 29.9 years (SD 3.6)
van Grieken et al. (2019) [29]	Mental health (stress)	The Supportive Parenting intervention	Quasi-experimental	Netherlands	N = 301 families; Intervention n = 124, mother M age = 31.0 years (SD 7.0), father M age = 34.0 years (SD 7.3), child M age = 7.6 months (SD 3.4), 47% child as girls; control n = 177, mother M age = 30.7 (SD 5.3), father M age = 32.6 years (SD 6.0), child M age = 5.1 months (SD 2.8).
van Horne et al. (2022) [52]	Mental health (depression)	Home visiting intervention	RCT	USA	N = 156 women; Intervention n = 72 women, M age = 30.65 years (SD 5.96); Control n = 46 women, M age = 29.4 years (SD 6.01).
Zapart et al. (2016) [23]	Women experiencing adversity (including mental health difficulties and domestic violence)	Sustained structured nurse home visiting program	Qualitative	Australia	N = 36 women, M age = 27.5 years (SD = 7.4).

Note. * = mental health intervention delivered via home visiting; ^a^ = domestic violence intervention. DOVE: Domestic Violence Enhanced Home Visitation Program; HoPES = Home Parenting Education and Support; IH-CBT = in-home cognitive-behavioural therapy; IMH-HV: infant mental health home visiting; M = mean; N = total number of participants; n = number of participants in subgroup; PFF = partnerships for families; RCT = randomised controlled trial; SPICE = special parent/infant care and enrichment; SD= Standard Deviation; TIES = Team for Infants Exposed to Substance abuse; VoorZorg = dutch nurse-family partnership; USA = United States of America.

### 4.1. Overview of Included Studies

Of the included studies, 23 were randomised control trials (RCTs), eight were quasi-experimental design studies, two were mixed-methods studies, and one was a qualitative study. Seventeen studies were conducted in the United States of America (USA), nine in Australia, four in the Netherlands, and Canada, the United Kingdom, New Zealand, and Japan each had one study. Sample sizes ranged from 16 to 739. Most studies focused on specific populations including parents with mentally ill health (e.g., depression, substance abuse, stress; 50%, *n* = 17), child maltreatment (20%, *n* = 7), or intimate partner violence populations (8%, *n* = 3). Several studies (20%, *n* = 7) focused on families considered to be “at risk” or “experiencing adversity” (i.e., families with multiple risk factors). One study included children experiencing emotional/behavioral problems and/or parent psychosocial risk (2%). The majority of programs were delivered by professionals (79%, *n* = 27).

### 4.2. Interventions

In the 34 studies, 25 different interventions were evaluated (see Supplementary Table S2 for an overview). Most programs were manualised but noted including flexibility based if required by families based on needs. The Michigan Model of Infant Mental Health Home Visiting (IMH-HV), In Home Cognitive Behavioural Therapy (IH-CBT; administered with families already participating in a home visiting program), and right@home were each evaluated in three studies. VoorZorg (Dutch version of Nurse Family Partnership), Sustained Structured Nurse Home Visiting Program, and the Hawaii Healthy Start Program were each evaluated in two studies. Numerous programs were evaluated in one study only, including: the Special Parent/Infant Care and Enrichment Program (SPICE), The Domestic Violence Enhanced Home Visitation Program (DOVE) Intervention, The Partnerships for Families (PFF) Mental Health Model, The Supportive Parenting Intervention, The Team for Infants Exposed to Substance abuse (TIES) Program, Early Start, Home Parenting Education and Support (HoPES), Healthy Families Arizona, Mums and Babies Program (Family First Partnership), Child FIRST, Cradle to Kinder, and Nurse Family Partnership. Six studies reported that a “home visiting” intervention, and one study indicated that “health visiting” intervention, was administered.

### 4.3. Intervention Components

The majority of interventions were implemented with caregivers of children agreed 0–24 months. Only 9 of the 34 studies included an intervention description that would allow for replicability in future studies, including IH-CBT [24,25,26], HoPES [30], Child FIRST [45], IMH-HV [28], The Supportive Parenting Intervention [29], and home visiting interventions by van Doesum et al. [51] and Bartu et al. [34]. The remaining studies had missing data on program components, program length, or both. Where information was available, Aslam and Kemp’s Aslam and Kemp [54] report was used as a guide to classify program components under seven main intervention types: counselling/psychological support; problem solving; child development; social support; parenting skills; parent infant interaction; and provision of resources, including information, equipment (such as safety equipment or books), and linking into community resources. From the studies that provided information on program length, the number of home visits ranged from 6–60 sessions, with the majority of sessions were delivered weekly (for long term interventions reported sessions were administered weekly and then as treatment progressed they were spaced out to fortnightly and/or monthly) and lasted between 15 min to 2.5 h (most reported session lengths between 60–90 min).

### 4.4. Outcomes

Given the focus of this review was on mental health, psychosocial outcomes, and parenting practices of caregivers, physical health outcomes (e.g., birth outcomes) are not reported. Outcomes were further divided into mental health (subgroups: depression, substance use, and stress); parenting practices (subgroups: practical parenting skills, maternal sensitivity/warmth/responsiveness, breastfeeding, reflective functioning, and the parent-child relationship/bond); and intimate partner violence outcomes. Table 3 provides a summary of outcomes. The outcomes are first presented as a narrative summary of both non-RCT and RCT studies, followed by meta-analysis results of RCT studies with available data. Where available, effect sizes and odds ratios have been reported. Effect sizes indicate the strength of the association between two variables. Effect sizes ranging from 0.2–0.49 indicate small effects, those ranging from 0.5–0.79 indicate medium effects, and those ranging from 0.8–1 indicate large effects. Odds ratios (OR) indicate how likely an outcome is in one group compared to another group with an OR of 1 indicating the odds are the same in both groups and ORs deviating from 1.0 indicating that odds are greater in one group compared to another.

Thirty-four studies reported on parent outcomes. Of these, 23 reported on maternal mental health outcomes [7,8,23,24,25,27,30,31,33,34,35,36,37,38,39,40,42,44,45,50,51,52,53], 14 reported on parenting practices [7,8,23,27,31,33,34,40,41,43,44,47,52,53], and five reported on intimate partner violence [32,38,39,46,49].

#### 4.4.1. Maternal Mental Health Outcomes

Of the 23 articles reporting on maternal mental health, the most measured outcomes were maternal depression (*n* = 12 articles) [7,24,25,33,38,39,40,42,44,45,50,51], substance use (*n* = 8) [8,34,35,37,38,39,44,53], and stress (*n* = 7) [24,25,30,35,36,40,45].

##### Depression

Seven of the 12 studies measuring maternal depression, five RCTs [7,40,42,45,50] and two quasi-experimental design [24,25] studies found a significant reduction in depressive symptoms in mothers who participated in a home visiting intervention. All studies used self-report measures of depression symptoms. RCTs were used to evaluate two “home visiting interventions” [40,50], the right@home intervention [7,42], and the child FIRST intervention [45]. The right@home intervention studies were longitudinal studies evaluating the same cohort. The quasi-experimental design studies were undertaken by the same team and evaluated IH-CBT, comparing home visiting interventions with CBT to home visiting interventions without CBT [24,25]. For the right@home intervention, the reduction in depression symptoms was maintained at the three- and five-year follow-up assessments with a small effect size (*d* = 0.20 for the 3 year follow up, *d* = 0.12 for the 5 year follow up) [7,42]. Fraser et al. [40] also completed a follow-up assessment, however, the observed reduction in depression symptoms in intervention primiparas (but not multiparae) women compared to control group was not maintained at the 12-month follow-up assessment.

**Figure 2:**
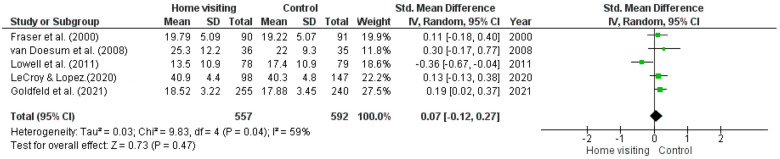
Forest plot for depression outcomes. Standardised mean differences are shown with 95% CIs. Green square = point estimate of intervention effect [40,42,44,45,51].

*Meta-analysis.* Five RCTs had sufficient data for a meta-analysis to be undertaken [40,42,44,45,51]. Studies with missing data or insufficient data required to calculate means (i.e., standard error, confidence intervals) were excluded from the meta-analysis. Substantial heterogeneity was observed among studies (Tau^2^ = 0.03; Chi^2^ = 9.83, *df* = 4, *p* = 0.04, *I*^2^ = 59%). The random effects model (standardised mean difference 0.07; 95% CI: −0.12, 0.27; *z* = 0.73, *p* = 0.47) found no significant difference between home visiting and control conditions (Figure 2).

##### Substance Use

Four of the eight studies measuring substance use, three RCTs [37,38,53] and one quasi-experimental design study [8], found significant reductions in substance use among mothers who participated in a home visiting intervention. All studies used self-report measures to assess substance use. The interventions evaluated included VoorZorg (focused on cigarette smoking), Hawaii Healthy Start Program (focused on illicit drug use and alcohol), TIES Program (focused on illicit drug use, alcohol, and tobacco), and the Nurse-Family Partnership Program (focused on cigarette smoking). A medium effect size (*ŋ_p_*^2^ = 0.09) was reported for the TIES Program [8]. Small to medium odds ratios were reported for the VoorZorg program (*OR* = 4.4 for average number of cigarettes smoked per day and *OR* = 1.6 for number of cigarettes smoked near baby). Data was not available for a meta-analysis.

##### Stress

Five of the six studies measuring stress, two RCTs [40,45] and three quasi-experimental [24,25,30] design studies, found a significant reduction in stress symptoms in mothers who participated in a home visiting intervention. All studies used self-report measures to assess stress levels. Fraser and colleagues [40] found a reduction in stress levels of primiparas (but not multiparae) intervention group women compared to control group women, however, these results were not maintained at a 12-month follow-up. Similarly, Lowell et al. [45] found a reduction in stress levels at six months and the results were not maintained at the 12-month follow-up. The quasi-experimental design studies evaluated the HoPES [30] and IH-CBT [24,25] interventions. The HoPES intervention reported a small effect size. The IH-CBT studies were undertaken by the same team and no effect sizes were reported.

**Figure 3:**
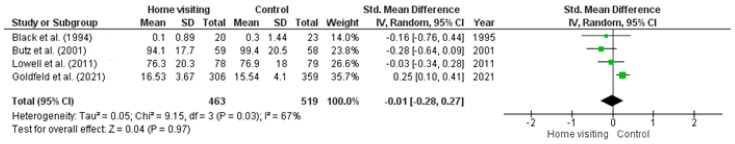
Forest plot for stress outcomes. Standardised mean differences are shown with 95% CIs. Green square = point estimate of intervention effect [35,36,42,45].

*Meta-analysis*. Four RCTs had sufficient data for a meta-analysis to be undertaken [35,36,42,45]. Studies with missing data or insufficient data required to calculate means (i.e., standard error, confidence intervals) were excluded from the meta-analysis. Substantial heterogeneity was observed among studies (Tau^2^ = 0.05; Chi^2^ = 9.15, *df* = 3, *p* = 0.03, *I*^2^ = 67%). The random effects model (standardised mean difference 0.01; 95% CI: −0.28, 0.27; *z* = 0.04, *p* = 0.97) found no significant difference between home visiting and control conditions (Figure 3).

#### 4.4.2. Parenting Practices

Of the 14 studies reporting on parenting practices, 12 reported on practical parenting skills (e.g., managing finances, maintaining contact with daycare providers), sense of competence, parenting self-efficacy, and/or views on motherhood [7,8,24,25,29,30,31,33,35,40,43,44]; five reported on maternal sensitivity/warmth/responsiveness [7,33,41,43,47]; four reported on breast feeding [34,43,44,53]; two reported on reflective functioning [27,49]; and one reported on parent-child bonding [52].

##### Practical Parenting Skills

Nine of the 12 studies reporting on practical parenting skills observed significantly improved parenting skills in women receiving a home visiting intervention. Of these studies, four were RCTs, four were quasi-experimental design studies, and one was a mixed-methods evaluation. All studies used self-report questionnaires to measure parenting skills. Two evaluated IH-CBT [24,25] and Healthy Families Arizona [44], HoPES [30], right@home [7], TIES [8], Cradle to Kinder [31], Kemp et al.’s [43] home visiting intervention, and Fraser et al.’s [40] home visiting intervention were evaluated in one study each. Both IH-CBT evaluations were undertaken by the same team, and both found that women had increased positive views of motherhood post-treatment. Effect sizes were not reported. LeCroy and Lopez [44] found that participants in the Healthy Family’s Arizona intervention, compared to control group, had significantly improved parenting practices (e.g., regular routines, reading to children) associated with moderate to large effect sizes (*d* range from 0.29 to 0.47). Giallo et al. [30] reported that women who took part in the HoPES intervention had increased parenting self-efficacy associated with a moderate effect size. Goldfeld et al. [7] reported improved parenting practices favoring women who took part in the right@home intervention, compared to control group, associated with small effect sizes (*d* ranged from 0.5 to 0.21). O’Malley et al. [8] reported a large effect size (*ŋ_p_*^2^ = 0.32) associated with improvements in parenting practices for women who participated in the TIES program. O’Donnell et al. [31] found significant improvements in parental capabilities with a medium effect size (*d* = 0.72) and caregivers reported perceived improvements in parenting skills and confidence. Kemp et al. [43] reported an improvement in emotional and verbal responsiveness during the first 24 months of their child’s life in women who were in the intervention group compared to control group with small effect sizes (*d* = 0.26). Fraser et al. [40] reported improved parenting sense of competence at six weeks postpartum for primiparas (but not multiparae) women in the intervention group. These results were not maintained at the 12-month follow-up. In addition to the studies that found positive outcomes of intervention, it is important to note that van Grieken et al.’ s [29] RCT found unchanged or worse outcomes, compared to baseline, for both intervention and control groups in regard to parenting skills (as well as self-sufficiency and resilience).

**Figure 4:**
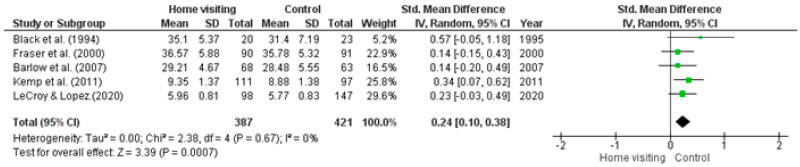
Forest plot for practical parenting skills outcomes. Standardised mean differences are shown with 95% CIs. Green square = point estimate of intervention effect [33,35,40,43,44].

*Meta-analysis*. Five RCTs had available data that could be used in a meta-analysis [35,36,40,43,44]. Heterogeneity was not significant (Tau^2^ = 0.00; Chi^2^ = 2.38, *df* = 4, *p* = 0.67, *I*^2^ = 0%). Studies with missing data or insufficient data required to calculate means (i.e., standard error, confidence intervals) were excluded from the meta-analysis. The random effects model (standardised mean difference 0.24; 95% CI: 0.10, 0.38; *z* = 3.39, *p* = 0.00) identified a significant difference between home visiting and control conditions, with results in favour of the control condition (Figure 4).

##### Maternal Sensitivity/Warmth/Responsiveness

Four of the five studies evaluating maternal sensitivity/warmth, all RCTs, found significant improvements in maternal sensitivity/warmth in women receiving home visiting interventions compared to control groups. Two studies evaluated the right@home intervention [7,41] and the Moms and Babies Program (Promoting First Relationships) [47] and Barlow et al. [33]’s health visiting intervention were evaluated in one study each. Three studies [7,33,41] used self-report questionnaires and one study used an observational measure [47] to assess maternal sensitivity/warmth. The right@home intervention studies were undertaken by the same team and reported results of a longitudinal study. Significant improvements in parental warmth were associated with a small effect size for the five-year follow-up assessment (*d* = 0.14) [7]. Oxford et al. [47] found that significant improvements in parental sensitivity were associated with a small effect size (*d* = 0.06) for women who experienced low distress and a large effect size (*d* = 0.63) for women who experienced high distress. Barlow et al. [33] did not report treatment effect sizes.

**Figure 5:**
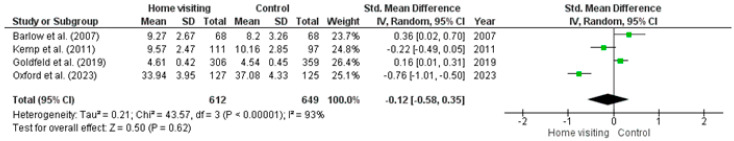
Forest plot for Maternal sensitivity/warmth/responsiveness outcomes. Standardised mean differences are shown with 95% CIs. Green square = point estimate of intervention effect [33,41,43,47].

*Meta-analysis*. Four RCTs had available data that could be used in a meta-analysis [33,41,43,47]. Studies with missing data or insufficient data required to calculate means (i.e., standard error, confidence intervals) were excluded from the meta-analysis. Results showed that there was significantly high heterogeneity (Tau^2^ = 0.21; Chi^2^ = 43.57, *df* = 3, *p* ≤ 0.001, *I*^2^ = 93%) among studies. The random effects model (standardised mean difference −0.12; 95% CI: −0.58, 0.35; *z* = 0.50, *p* = 0.62) found a non-significant difference between home visiting and control conditions (Figure 5).

##### Breast Feeding

Three of the four studies found significant improvements in breast feeding duration as a result of participating in a home visiting intervention, all were RCTs and utilized self-report questionnaires [43,44,53]. The interventions implemented included Healthy Families Arizona, VoorZorg, and Kemp et al. [43]’s home visiting intervention. Reported effect sizes for Healthy Families Arizona (*d* = 0.29) and Kemp et al. [43]’s home visiting intervention (*d* = 0.49) were in the medium range. An odds ratio was reported for the VoorZorg intervention which indicated a small to medium association between the intervention and breast feeding (*OR* = 2.6). Data was not available for meta-analysis.

##### Reflective Functioning

Both studies that provided outcomes on parental reflective functioning evaluated IMH-HV, used a quasi-experimental design, and were undertaken by the same team. Stacks et al. [27] used an observational procedure, while Stacks et al. [28] used a clinician administered structured interview, to assess reflective functioning. Results of both studies showed significant improvements in parental reflective functioning post-treatment. Stacks et al. [27] reported an associated between intervention and reflective functioning improvement with a moderate effect size (*d* = 0.43).

##### Parent-Child Bonding/Relationship

Only one study evaluated mothers’ perceptions of her bond/relationship with her baby [52]. The study utilised an RCT to evaluate a home visiting intervention developed by van Horne et al. [52] and used self-report measures to measure the parent-child bond/relationship. No significant improvements were observed in the Parent-child bond/relationship post treatment compared to control group.

#### 4.4.3. Intimate Partner Violence

Three out of five studies evaluating the impact of home visiting interventions on intimate partner violence reported significant reductions in intimate partner violence for women who participated in the intervention group compared to women in the control group. All studies were RCTs and used the Conflict Tactics Scale, a self-report measure, to measure intimate partner violence. Interventions implemented included the DOVE intervention [49], Mejdoubi et al. [46]’s home visiting intervention and Hawaii Healthy Start Program [32]. Sharps et al. [49] found that the DOVE intervention was associated with a moderate effect size (*d* = 0.50). An odds ratio was reported for Mejdoubi et al. [46]’s home visiting intervention which indicated a small association between intervention and reduced intimate partner violence (*OR* = 0.48). The initial association between the Hawaii Healthy Start Program and lower intimate partner violence was not maintained long term. Data was not available for meta-analysis.

**Table 3:** (**a**) RCT Summary Table. (**b**): Non-RCT Summary table.

**(a)** RCT studies						
**Outcome**	**Citation**	**Target Problem**	**Intervention**	**Results**		**Intervention Components**
				Sig.	Non. Sig.	
Maternal mental health	Barlow et al. (2007) [33]	Mental health (depression)	Health visiting intervention		x	Parent-infant interaction
	Bartu et al. (2006) [34]	Mental health (substance use)	Home visiting intervention		x	Social support Child development Parenting skills Parent-infant interaction Provision of resources
	Black et al. (1994) [35]	Mental health (substance use)	SPICE		x	Social support Parenting skills and child development Parent-infant interaction Provision of resources
	Butz et al. (2001) [36]	Mental health (substance use)	Home visiting intervention		x	Parenting skills Child development Parent-infant interaction Provision of resources
	Catherine et al. (2020) [37]	Mental health (substance use)	Nurse-Family Partnership	x		Social support
	Duggan et al. (2004) [38]	Child maltreatment	Hawaii Healthy Start Program	x		Social supports Parenting skills
	Fergusson et al. (2005) [39]	At risk families (including mental health difficulties and domestic violence)	Early start		x	Social supports Problem solving Parenting skills
	Fraser et al. (2000) [40]	Child maltreatment	Home visiting intervention	x		Social supports Provision of resources
	Goldfeld et al. (2021) [42]	At risk women (including mental health difficulties)	right@home	x		Parenting skills Parent-infant interaction Provision of resources
	Goldfeld et al. (2022) [7]	Women experiencing adversity (including mental health difficulties)	right@home	x		Parenting skills Parent-infant interaction Provision of resources
	LeCroy & Lopez. (2020) [44]	Mental health	Healthy Families Arizona	x		Parent-infant interaction
	Lowell et al. (2011) [45]	Child emotional/behavioural problems and/or parent psychosocial risk	Child FIRST	x		Counselling or Psychological Support
	Mejdoubi et al. (2014) [53]	At risk women (including substance use and domestic violence)	VoorZorg	x		Parenting skills and child development Parent infant interaction Social supports
	Tamaki (2008) [50]	Mental health (depression)	Home visiting intervention	x		Counselling or Psychological support Parenting skills and child development Parent-infant interaction Problem solving Social supports Provision of resources
	van Doesum et al. (2008) [51]	Mental health (depression)	Home visiting intervention		x	Parenting skills and child development Parent-infant interaction Problem solving Provision of resources
	van Horne et al. (2022) [52]	Mental health (depression)	Home visiting intervention		x	Counselling or Psychological support Parenting skills and child development Parent-infant interaction Problem solving Provision of resources
Parenting practices	Barlow et al. (2007) [33]	Mental health (depression)	Health visiting intervention	x		Parent-infant interaction
	Bartu et al. (2006) [34]	Mental health (substance use)	Home visiting intervention		x	Social support Child development Parenting skills Parent-infant interaction Provision of resources
	Fraser et al. (2000) [40]	Child maltreatment	Home visiting intervention	x		Social supports Provision of resources
	Goldfeld et al. (2019) [41]	Women experiencing adversity (including mental health difficulties)	right@home	x		Parenting skills Parent-infant interaction Provision of resources
	Goldfeld et al. (2022) [7]	Women experiencing adversity (including mental health difficulties)	right@home	x		Parenting skills Parent-infant interaction Provision of resources
	Kemp et al. (2011) [43]	Women experiencing adversity (including mental health difficulties and domestic violence)	Sustained structured nurse home visiting program	x		Parenting skills Parent-infant interaction Provision of resources
	LeCroy & Lopez. (2020) [44]	Mental health	Healthy Families Arizona	x		Parent-infant interaction
	Mejdoubi et al. (2014) [53]	At risk women (including substance use and domestic violence)	VoorZorg	x		Parenting skills and child development Parent infant interaction Social supports
	Oxford et al. (2023) [47]	Mental health	Moms and Babies Program (Promoting First Relationships)	x		Parenting skills and child development Parent infant interaction
	van Horne et al. (2022) [52]	Mental health (depression)	Home visiting intervention		x	Counselling or Psychological support Parenting skills and child development Parent-infant interaction Problem solving Provision of resources
Intimate partner violence	Bair-Merritt et al. (2010) [32]	Partner violence	Hawaii Healthy Start Program	x		Social support Problem solving Parenting skills Parent-infant interaction Provision of resources
	Duggan et al. (2004) [38]	Child maltreatment	Hawaii Healthy Start Program		x	Social supports Parenting skills
	Fergusson et al. (2005) [39]	At risk families (including mental health difficulties and domestic violence)	Early start		x	Social supports Problem solving Parenting skills
	Mejdoubi et al. (2013) [46]	Partner violence	VoorZorg	x		Parenting skills and child development Parent infant interaction Social supports
	Sharps et al. (2016) [49]	Partner violence	DOVE	x		Social support Parenting skills and child development
**(b)** Non-RCT studies						
**Outcomes**	**Citation**	**Target Problem**	**Intervention**	**Results**		**Intervention Components**
				Sig.	Non. Sig.	
Maternal mental health	Ammerman et al. (2011) [25]	Mental health (depression)	IH-CBT	x		Counselling or Psychological Support
	Ammerman et al. (2005) [24]	Mental health (depression)	IH-CBT	x		Counselling or Psychological Support
	Giallo et al. (2021) [30]	Child maltreatment	HoPES	x		Parenting skills Parent-infant interaction
	O’Donnell (2023) [31]	Families with multiple risk factors (e.g., family violence, substance use, mental health concerns, Child Protection involvement)	Cradle to Kinder	x		Counselling or Psychological Support Parenting skills and child development Parent infant interaction Social supports Provision of resources
	O’Malley et al. (2021) [8]	Mental health (substance use)	TIES	x		Parenting skills and child development Parent infant interaction Provision of resources
	Stacks et al. (2019) [27]	Child maltreatment	Michigan Model of IMH-HV	x		Social support Parenting skills and child development Parent-infant interaction Provision of resources
Parenting practices	Ammerman et al. (2011) [25]	Mental health (depression)	IH-CBT	x		Counselling or Psychological Support
	Ammerman et al. (2005) [24]	Mental health (depression)	IH-CBT	x		Counselling or Psychological Support
	Giallo et al. (2021) [30]	Child maltreatment	HoPES	x		
	O’Donnell (2023) [31]	Families with multiple risk factors (e.g., family violence, substance use, mental health concerns, Child Protection involvement)	Cradle to Kinder	x		Counselling or Psychological Support Parenting skills and child development Parent infant interaction Social supports Provision of resources
	O’Malley et al. (2021) [8]	Mental health (substance use)	TIES	x		Parenting skills and child development Parent infant interaction Provision of resources
	Stacks et al. (2022) [28]	Mental health	Michigan Model of IMH-HV		x	Counselling or Psychological Support Social support Parent-infant interaction Parenting skills and child development
	Stacks et al. (2019) [27]	Child maltreatment	Michigan Model of IMH-HV	x		Social support Parenting skills and child development Parent-infant interaction Provision of resources

Note. DOVE: Domestic Violence Enhanced Home Visitation Program; IMH-HV: Infant Mental Health-Home Visiting; SPICE: Sustained Program for Improving Childhood Education. HoPES = Healthy Parenting, Healthy Families. IH-CBT = In-Home Cognitive Behavioral Therapy. Michigan Model of IMH-HV = Michigan Model of Infant Mental Health-Home Visiting. TIES = Team for Infants Exposed to Substance Abuse.

## 5. Discussion

This review evaluated the impact of home visiting interventions administered by professionals/paraprofessionals on caregiver outcomes. The review evaluated 34 studies to determine the impact of home visiting interventions on caregivers with high vulnerability/complex needs and young children. Overall, the review found that home visiting interventions improved a range of caregiver outcomes, although meta-analysis did not reach significance.

A total of 25 different interventions were evaluated, primarily using RCTs. Most studies included large samples and indicated positive findings across a range of outcomes: 50–80% of studies reporting on parent mental health, 75–100% reporting on parenting practices, and 60% reporting on intimate partner violence found significant improvements in outcomes. Meta-analysis effect sizes ranged from −0.01 to 0.24. Only the meta-analysis evaluating practical parenting skills was, however, significantly different from 0, with a small to medium effect size; though, results favoured the control group. Meta-analysis results need to be interpreted with caution due to high heterogeneity and the fact that most studies did not have available data that could be included in the meta-analysis.

When results of all studies are taken into consideration, findings suggest that home visiting interventions can lead to improvements in parenting outcomes. The variation in programs delivered along with a large number of studies not providing information on intervention time/dosage meant that broad stroke evaluations were unable to identify clear patterns regarding which program components led to an effective intervention and which led to an ineffective intervention. The literature was limited by the fact that only four studies collected data on fathers [29,30,31,44] and only two studies segregated some outcomes by parent gender [30,31]. Thus, the conclusions drawn from this body of work primarily pertain to mothers/female caregivers. Given that a commonly cited benefit of home visiting interventions is that the whole family can be involved in the intervention [10,55,56], it was surprising that more studies did not include fathers. While surprising, this was not entirely unexpected given the challenges associated with engaging fathers/male caregivers in health services [57]. A recent systematic review [57] on barriers associated with health service access by fathers/male caregivers found that both individual factors (e.g., narrow views of masculinity) and health service factors (e.g., service focus on mothers) are at play when it comes to fathers/male caregivers engaging with health services. The review identified several ways health services could improve father/male caregiver engagement, including providing father-specific resources/support and improving health professionals’ knowledge/confidence regarding working with fathers. Future research should aim to increase father/male caregiver participation to identify the impact of home visiting interventions on male caregivers, and the family as a whole.

### 5.1. Clinical Considerations

A number of home visiting interventions aimed at improving outcomes in caregivers who have young children and experience high vulnerability/complex needs were identified. While most interventions were found to be effective at improving at least one caregiver outcome and the majority of studies used the gold standard RCT to evaluate outcomes, a significant limitation of the literature was that most interventions were only evaluated in one study. IH-CBT, right@home, VoorZorg, and IMH-HV were the only interventions evaluated in two or more studies. The right@home studies reported data on one cohort and had a high risk of bias. Furthermore, IH-CBT, right@home, and VoorZorg studies were all evaluated by one team of researchers, increasing the risk of bias. The study heterogeneity and missing data did not allow for comparisons between effective and non-effective innervations. Though broad stroke evaluation did not identify differences in components between effective and non-effective interventions. Furthermore, the majority of studies focused on maternal caregivers, thus the impact of home visiting interventions on paternal caregivers and the family as a whole unit remains unclear. Thus, taking into consideration the available literature, no one intervention appears suitable to address all the varied needs of caregivers with high vulnerability and complex needs. When determining the appropriate intervention for the population, the needs of that population need to be considered. For example, right@home showed long term improvements in caregiver depression, HoPES had the greatest evidence for improving caregiver stress, while the DOVE intervention had the strongest evidence for reducing intimate partner violence.

### 5.2. Strengths and Limitations

The use of a systematic review strategy with broad inclusion criteria to increase the chances of including all relevant literature; trial registration; two reviewers reviewing all included studies; and detailed quality assessments are significant strengths of this review. The review also had several limitations. First, although common practice with systematic reviews, the review was restricted to studies written in the English language, limiting the generalisability of findings. Second, the inclusion of studies conducted exclusively in high-income, predominantly English-speaking countries also compromised the generalisability of the results. Third, only programs delivered by professionals/paraprofessionals were reported, the inclusion of studies in which home visiting interventions were administered by volunteer home visitors may have led to a different pattern in results. Fourth, high heterogeneity among studies was also identified. The cause of heterogeneity remains unclear and could have been the result of several factors including the fact that different interventions were administered, the duration of follow-up across studies was inconsistent, the inclusion/exclusion criteria was variable, and there was variability in outcome measures. Subgroup analysis of intervention time/dosage were not possible due to missing data. Fifth, inter-rater reliability for full-text screening was lower than 80% agreement, which is generally considered acceptable. The lower agreement on full text screenings is attributed to varying definitions of what constitutes a family with high vulnerability and/or complex needs as well as the limited information provided in some articles regarding the qualifications home visitors. Sixth, most studies used self-report measures to evaluate intervention impacts. This increases the chances of biases such as recall bias and social desirability bias around sensitive topics such as intimate partner violence and mental health challenges.

## 6. Conclusions

This review found that home visiting interventions delivered to caregivers who have young children and experience high vulnerability/complex needs can result in improvements in a variety of caregiver outcomes. Whilst meta-analysis of RCTs did not reach significance, positive treatment effects for one or more outcomes were observed for most home visiting programs. Further research on each intervention, with more diverse samples, and using a range of measures is needed.

## Data Availability

The datasets used and/or analysed during the current study are available from the corresponding author on reasonable request.

## Supplementary Materials

The following supporting information can be downloaded at: https://www.mdpi.com/article/10.3390/ijerph23010063/s1, Supplementary Table S1: Search strategy and search terms; Supplementary Table S2: Intervention Overview; Appendix S1: PRISMA checklist.

## Abbreviations

DOVE	The Domestic Violence Enhanced Home Visitation Program
HomVEE	Home Visiting Evidence of Effectiveness
HoPES	Program, Early Start, Home Parenting Education and Support
IH-CBT	In Home Cognitive Behavioural Therapy
IMH-HV	The Michigan Model of Infant Mental Health Home Visiting
MMAT	Mixed Methods Appraisal Tool
PFF	The Partnerships for Families
PRISMA	Preferred Reporting Items for Systematic Reviews and Meta-Analysis
RCT	Randomised control trial
SPICE	Special Parent/Infant Care and Enrichment Program
TIES	The Team for Infants Exposed to Substance abuse
USA	United States of America
