# Supplementary material for: Home Visiting Interventions and Their Impact on Mental Health, Psychosocial, and Parenting Practice Outcomes of Vulnerable Caregivers: A Systematic Review and Meta-Analysis
Source: Int J Environ Res Public Health. 2025 Dec 31;23(1):63. doi: 10.3390/ijerph23010063 (PMC12841137; doi:10.3390/ijerph23010063)
Supplement: Supplementary file 1 [file ijerph-23-00063-s001.zip › ijerph-3938192-supplementary.pdf]

Supplementary Tables

Supplementary Table S1

Search strategy and search terms

Data base	Date searched	Search Terms
PsychInfo	11.08.2023	APA PsycInfo <1806 to July Week 5 2023><br>1 Home Visiting Programs/ 2019<br>2 Intervention/ or "Psychotherapy (Individual)"/ or Program/ or Prevention/ or Support.mp. [mp=title, abstract, heading word, table of contents, key concepts, original title, tests & measures, mesh word] 699077<br>3 Postnatal/ or Perinatal/ or Antenatal/ or Postpartum/ or Parent/ or Mother/ or Father/ or Caregiver.mp. [mp=title, abstract, heading word, table of contents, key concepts, original title, tests & measures, mesh word] 38145<br>4 Mental Health/ or Substance abuse problem.mp. or Domestic Violence/ or Child Protective Services.mp. 104822<br>5 "Evaluation (Treatment Effectiveness)"/ or (Psychosocial Outcomes/ or Treatment Outcomes/ or "Outcomes (Treatment)"/ or Health Outcomes/) 71637<br>6 1 and 2 and 3 and 4 and 5 1
Scopus	11.08.2023	(TITLE-ABS-KEY (home AND visit) AND TITLE-ABS-KEY (intervention OR program OR therapy OR prevention OR support) AND TITLE-ABS-KEY (postnatal OR perinatal OR antenatal OR postpartum OR parent OR mother OR father OR caregiver) AND TITLE-ABS-KEY ("Mental health" OR drug OR alcohol OR substance OR "Domestic Violence" OR "Child protection") AND TITLE-ABS-KEY (evaluation OR effectiveness OR outcome)) AND (LIMIT-TO (DOCTYPE , "ar")) AND (LIMIT-TO (LANGUAGE , "English")) AND (LIMIT-TO (EXACTKEYWORD , "Human") OR LIMIT-TO (EXACTKEYWORD , "Child") OR LIMIT-TO (EXACTKEYWORD , "Infant") OR EXCLUDE (EXACTKEYWORD , "Aged")) AND (LIMIT-TO (PUBSTAGE , "final"))
Embase	11.08.2023	# Searches Results<br>1 home visit/ 5033<br>2 early intervention/ or Intervention.mp. or intervention study/ or psychosocial intervention/ 1284369

		3 program evaluation/ or program feasibility/ or program sustainability/ or health program/ or program impact/ or education program/ or program effectiveness/ or Program.mp. or program efficacy/ 1115576<br>4 Therapy.mp. 9766130<br>5 prevention/ 322790<br>6 Support.mp. 1757752<br>7 2 or 3 or 4 or 5 or 6 12639531<br>8 (Postnatal or Perinatal or Antenatal or Postpartum or Parent or Mother or Father or Caregiver).mp. [mp=title, abstract, heading word, drug trade name, original title, device manufacturer, drug manufacturer, device trade name, keyword heading word, floating subheading word, candidate term word] 1099861<br>9 mental health/ 210738<br>10 (Drug or Alcohol or Substance).mp. [mp=title, abstract, heading word, drug trade name, original title, device manufacturer, drug manufacturer, device trade name, keyword heading word, floating subheading word, candidate term word] 14113243<br>11 domestic violence/ 11606<br>12 child welfare/ or child protection/ 20410<br>13 9 or 10 or 11 or 12 14299652<br>14 (Evaluation or effectiveness or Outcome).mp. [mp=title, abstract, heading word, drug trade name, original title, device manufacturer, drug manufacturer, device trade name, keyword heading word, floating subheading word, candidate term word] 6312165<br>15 1 and 7 and 8 and 13 and 14 122<br>16 limit 15 to (full text and human and english language and "remove preprint records" and article and child) 5
CINAHL	11.08.2023	"(home visits or home visiting or house calls or home care or home health care or home based) AND (interventions or strategies or best practices or treatment or therapy or program or management or support or prevention) AND (Postnatal OR Perinatal OR Antenatal OR Postpartum OR Parent OR Mother OR Father OR Caregiver) AND (mental health or mental illness or mental disorder or psychiatric illness) OR (drug addiction or drug abuse or substance abuse) OR (domestic violence or domestic abuse or intimate partner violence) AND (evaluation or assessment or measurement or analysis) OR (effectiveness or efficacy or effective or success or outcome)"
PubMed	11.08.2023	((((home visit) AND (Intervention OR Program OR Therapy OR Prevention OR Support)) AND (Postnatal OR Perinatal OR Antenatal OR Postpartum OR Parent OR Mother OR Father OR Caregiver)) AND ("Mental health" OR Drug OR Alcohol OR Substance OR "Domestic Violence" OR "Child protection")) AND (Evaluation OR effectiveness OR Outcome) Filters applied: Full text, Case Reports, Classical Article, Clinical Study, Clinical Trial, Evaluation Study,

		Observational Study, Randomized Controlled Trial, Humans, English, Newborn: birth-1 month, Infant: birth-23 months, Infant: 1-23 months, Preschool Child: 2-5 years, Exclude preprints

Supplementary Table S2

Intervention Overview

Citation	Intervention	Program deliverers	Risk factors for program entry	Classification of Key Program Components	Training required for program delivery	Number of sessions	Session length (minutes)	Session frequency	Target child age group
Ammerman et al. (2005)	IH-CBT	Social worker, nurse, or degree in related discipline	Major Depressive Disorder	Counselling or Psychological Support	Y	15	60	Weekly	Pregnancy and post-birth
Ammerman et al. (2011)	IH-CBT	Social worker	Major Depressive Disorder	Counselling or Psychological Support	Y	15	60	Weekly	20 weeks gestation - 3 months postpartum
Ammerman et al. (2012)	IH-CBT	Social worker	Major Depressive Disorder	Counselling or Psychological Support	Y	15	60	Weekly	3 months post-partum
Bair-Merritt et al. (2010)	Hawaii Healthy Start Program	Paraprofessional	Parents of infant who was at high risk for maltreatment	Social support<br>Problem solving<br>Parenting skills<br>Parent-infant interaction<br>Provision of resources	NP	Mean 13.6 visits in the first year	NP	Weekly visits initially, then spread out	0- 3 years
Barlow et al. (2007)	Health visiting intervention	Health visitors	Mental health problems	Parent-infant interaction	Y	NP	NP	NP	6-12 months
Bartu et al. (2006)	Home visiting intervention	Midwife	Illicit drug use	Social support<br>Child development<br>Parenting skills<br>Parent-infant interaction<br>Provision of resources	NP	8	60-120	Weekly/m onthly	35–40 weeks gestation

Black et al. (1994)	SPICE	Nures	Prenatal cocaine or heroin use	Social support<br>Parenting skills and child development<br>Parent-infant interaction<br>Provision of resources	NP	Not provided	60	Bi-weely	Pregnancy - 18 months
Butz et al. (2001)	Home visiting intervention	Nurse	Substance use	Parenting skills<br>Child development<br>Parent-infant interaction<br>Provision of resources	Y	16	Not provided	Not provided	0-18 months
Catherine et al. (2020)	Nurse-Family Partnership	Paraprofessional	Perinatal substance use	Social support	NP	14-50	NP	NP	0-2 years
Duggan et al. (2004)	Hawaii Healthy Start	Paraprofessional	Parents of infant who was at high risk for maltreatment	Social supports<br>Parenting skills	Y	NP	NP	NP	NP
Fergusson et al. (2005)	Early Start	Nurse	Parental substance use and family violence.	Social supports<br>Problem solving<br>Parenting skills	NP	NP	NP	NP	0-3 years
Fraser et al. (2000)	Home visiting intervention	Nurses, social workers, parental aids, paediatrician	A history of mental health disorder (either parent), alcohol or drug abuse, and/or domestic violence	Social supports<br>Provision of resources	Y	NP	20-60	Weekly, fortnight, monthly	0-12 months
Giallo et al. (2021)	HoPES	Social worker	Risk factors for child maltreatment	Parenting skills<br>Parent-infant interaction	NP	14	150	2 times per week	0-4 years
Goldfeld et al. (2019, 2021, 2022)	right@home	Nurse, social care practitioner	Current smoker, experiencing stress, anxiety, or difficulty coping.	Parenting skills<br>Parent-infant interaction<br>Provision of resources	Y	25	60-90	NP	0- 2 years
Kemp et al. (2011), Zapart et al. (2015)	Sustained structured nurse home	Nurse	Stressors expereinced in pregnancy that could negatively	Parenting skills<br>Parent-infant interaction<br>Provision of resources	NP	0-52	60-90	NP	0-2 years

	visiting program		impact on maternal and child outcomes.						
LeCroy & Lopez.(2020)	Healthy Families Arizona	Paraprofessional	Parent childhood history of abuse or neglect, potential for violence, and a history of mental illness, criminality, and drug abuse.	Parent-infant interaction	Y	NP	NP	Weekly and then tapered off as families made progress	Not provided
Lowell et al. (2011)	Child FIRST	mental health clinicians	Child social-emotional ⁄ behavioural problems and ⁄ or the parent psychosocial risk	Counselling or Psychological Support	NP	22.1 (average)	45–90	Weekly	6-36 months
Mejdoubi et al. (2013)	VoorZorg	Nurse	A history or present situation of domestic violence, psychosocial symptoms, alcohol and/or drug use.	Parenting skills and child development<br>Parent infant interaction<br>Social supports	NP	40-60	NP	NP	0-2 years
Mejdoubi et al. (2014)	VoorZorg	Nures	A history or present situation of domestic violence, psychosocial symptoms, alcohol and/or drug use.	Parenting skills and child development<br>Parent infant interaction<br>Social supports	Y	20	NP	Weekly	0-2 years

O'Donnell (20230	Cradle to Kinder	Family support practitioners (i.e., key worker), maternal and child health nurses and early parenting workers	Families in which the mother was pregnant or within the first six weeks postpartum and were the subject of an unborn report made to Child Protection regarding the welfare of their child or the primary caregiver exhibited several characteristics placing them at-risk of child removal (e. g., poverty, homelessness, alcohol and other drug use, mental ill health, family violence)	Counselling or Psychological Support<br>Parenting skills and child development<br>Parent infant interaction<br>Social supports<br>Provision of resources	Y	NP (families received 558 to 1311 hours of interventi on)	NP	Dependent on needs (average = twice weekly for first 12 months then once weekly)	
O'Malley et al. (2021)	TIES	Social worker	Maternal substance use	Parenting skills and child development<br>Parent infant interaction<br>Provision of resources	Y	NP	NP	NP	0-2 years
Oxford et al. (2023)	Moms and Babies Program (Promoting First Relationships)	Mental health service providers	Mental health concerns during pregnancy	Parenting skills and child development<br>Parent infant interaction	Y	weekly	NP	NP	6-12 weeks

Reuter et al. (2016)	PFF	Therapist	Pregnant women with risk factors for child maltreatment such as domestic violence, maternal substance abuse, maternal depression, and/or other mental health concerns	Counselling or Psychological Support<br>Parenting skills and child development<br>Social supports<br>Provision of resources	Y	NP	90	Weekly	Children under 5
Rosenblum et al. (2020)	Michigan Model of IMH-HV	Clinician	Possible depression diagnosis	Social support<br>Parenting skills and child development<br>Parent-infant interaction<br>Provision of resources	Y	NP	NP	NP	0-24 months
Sharps et al. (2016)	DOVE	Nurse, community health worker	Perinatal intimate partner violence	Social support<br>Parenting skills and child development	Y	6	15-25	NP	0-12 months
Stacks et al. (2019)	Michigan Model of IMH-HV	Therapist	Child welfare concerns	Social support<br>Parenting skills and child development<br>Parent-infant interaction<br>Provision of resources	Y	NP		Weekly	0-3 years
Stacks et al. (2022)	Michigan Model of IMH-HV	Mental health therapist	Psychosocial risk	Counselling or Psychological Support<br>Social support<br>Parent-infant interaction<br>Parenting skills and child development	Y	20 - 48	60-120	Weekly	Pregnancy- 3 years
Tamaki (2008)	Home visiting intervention	Nurse	Possible depression diagnosis	Counselling or Psychological support<br>Parenting skills and child development<br>Parent-infant interaction<br>Problem solving<br>Social supports<br>Provision of resources	Y	4	NP	NP	0- 4 months

van Doesum et al. (2008).	Home visiting intervention	Psychologists, psychiatrist	Major depressive episode or dysthymia and/or exhibited elevated levels of depressive symptoms	Parenting skills and child development<br>Parent-infant interaction<br>Problem solving<br>Provision of resources	Y	8 to 10	60-90	Weekly / biweekly	0-12 months
van Grieken et al. (2019)	The Supportive Parenting intervention	Nurse	Parents At Risk for child Abuse and Neglect	Parenting skills<br>Parent-infant interaction<br>Social supports	Y	6	90	Spread out	0- 18 months
van Horne et al. (2022)	Home visiting intervention	Social worker	Mild to moderate depression symptoms	Counselling or Psychological support<br>Parenting skills and child development<br>Parent-infant interaction<br>Problem solving<br>Provision of resources	Y	5	30-60	NP	$\leq$ 4 months

Note. Information included in this table was collected from included articles, protocol papers when available, and intervention websites when available. ACE: Adverse Childhood Experiences; DOVE: Domestic Violence Enhanced Home Visitation Program; HOPE: Home Parenting Education And Support; IH-CBT: In-Home Cognitive-Behavioural Therapy; IMH-HV: Infant Mental Health Home Visiting; NP: Information Not Provided; PFF: Partnerships For Families; SPICE: Special Parent/Infant Care And Enrichment; TIES: Team For Infants Exposed To Substance Abuse; Voorzorg: A Dutch Nurse-Family Partnership; Y: Yes.

Appendix

Appendix 1.
PRISMA checklist

Section and Topic	Item #	Checklist item	Location where item is reported
**TITLE**			
Title	1	Identify the report as a systematic review.	Title
**ABSTRACT**			
Abstract	2	See the PRISMA 2020 for Abstracts checklist.	Abstract
**INTRODUCTION**			
Rationale	3	Describe the rationale for the review in the context of existing knowledge.	Introduction
Objectives	4	Provide an explicit statement of the objective(s) or question(s) the review addresses.	Introduction
**METHODS**			
Eligibility criteria	5	Specify the inclusion and exclusion criteria for the review and how studies were grouped for the syntheses.	Method-Inclusion and Exclusion Criteria
Information sources	6	Specify all databases, registers, websites, organisations, reference lists and other sources searched or consulted to identify studies. Specify the date when each source was last searched or consulted.	Method-Search Strategy e
hSearch strategy	7	Present the full search strategies for all databases, registers and websites, including any filters and limits used.	Supplementary Table 1

Section and Topic	Item #	Checklist item	Location where item is reported
Selection process	8	Specify the methods used to decide whether a study met the inclusion criteria of the review, including how many reviewers screened each record and each report retrieved, whether they worked independently, and if applicable, details of automation tools used in the process.	Results
Data collection process	9	Specify the methods used to collect data from reports, including how many reviewers collected data from each report, whether they worked independently, any processes for obtaining or confirming data from study investigators, and if applicable, details of automation tools used in the process.	Results
Data items	10a	List and define all outcomes for which data were sought. Specify whether all results that were compatible with each outcome domain in each study were sought (e.g. for all measures, time points, analyses), and if not, the methods used to decide which results to collect.	Methods, results
	10b	List and define all other variables for which data were sought (e.g. participant and intervention characteristics, funding sources). Describe any assumptions made about any missing or unclear information.	Methods, results
Study risk of bias assessment	11	Specify the methods used to assess risk of bias in the included studies, including details of the tool(s) used, how many reviewers assessed each study and whether they worked independently, and if applicable, details of automation tools used in the process.	Methods, results
Effect measures	12	Specify for each outcome the effect measure(s) (e.g. risk ratio, mean difference) used in the synthesis or presentation of results.	Method
Synthesis methods	13a	Describe the processes used to decide which studies were eligible for each synthesis (e.g. tabulating the study intervention characteristics and comparing against the planned groups for each synthesis (item #5)).	Methods, results
	13b	Describe any methods required to prepare the data for presentation or synthesis, such as handling of missing summary statistics, or data conversions.	Methods
	13c	Describe any methods used to tabulate or visually display results of individual studies and syntheses.	Results
	13d	Describe any methods used to synthesize results and provide a rationale for the choice(s). If meta-analysis was performed, describe the model(s), method(s) to identify the presence and extent of statistical heterogeneity, and software package(s) used.	Aims

Section and Topic	Item #	Checklist item	Location where item is reported
	13e	Describe any methods used to explore possible causes of heterogeneity among study results (e.g. subgroup analysis, meta-regression).	Discussion
	13f	Describe any sensitivity analyses conducted to assess robustness of the synthesized results.	N/A
Reporting bias assessment	14	Describe any methods used to assess risk of bias due to missing results in a synthesis (arising from reporting biases).	Methods, results
Certainty assessment	15	Describe any methods used to assess certainty (or confidence) in the body of evidence for an outcome.	Results- risk of bias assessments
**RESULTS**			
Study selection	16a	Describe the results of the search and selection process, from the number of records identified in the search to the number of studies included in the review, ideally using a flow diagram.	Results
	16b	Cite studies that might appear to meet the inclusion criteria, but which were excluded, and explain why they were excluded.	Results
Study characteristics	17	Cite each included study and present its characteristics.	Results
Risk of bias in studies	18	Present assessments of risk of bias for each included study.	Results
Results of individual studies	19	For all outcomes, present, for each study: (a) summary statistics for each group (where appropriate) and (b) an effect estimate and its precision (e.g. confidence/credible interval), ideally using structured tables or plots.	Results
Results of syntheses	20a	For each synthesis, briefly summarise the characteristics and risk of bias among contributing studies.	Results
	20b	Present results of all statistical syntheses conducted. If meta-analysis was done, present for each the summary estimate and its precision (e.g. confidence/credible interval) and measures of statistical heterogeneity. If comparing groups, describe the direction of the effect.	Results
	20c	Present results of all investigations of possible causes of heterogeneity among study results.	Discussion

Section and Topic	Item #	Checklist item	Location where item is reported
	20d	Present results of all sensitivity analyses conducted to assess the robustness of the synthesized results.	N/A
Reporting biases	21	Present assessments of risk of bias due to missing results (arising from reporting biases) for each synthesis assessed.	Results, Discussion
Certainty of evidence	22	Present assessments of certainty (or confidence) in the body of evidence for each outcome assessed.	Results, discussion
**DISCUSSION**			
Discussion	23a	Provide a general interpretation of the results in the context of other evidence.	Discussion
	23b	Discuss any limitations of the evidence included in the review.	Discussion
	23c	Discuss any limitations of the review processes used.	Discussion
	23d	Discuss implications of the results for practice, policy, and future research.	Discussion
**OTHER INFORMATION**			
Registration and protocol	24a	Provide registration information for the review, including register name and registration number, or state that the review was not registered.	Method
	24b	Indicate where the review protocol can be accessed, or state that a protocol was not prepared.	Method
	24c	Describe and explain any amendments to information provided at registration or in the protocol.	Method
Support	25	Describe sources of financial or non-financial support for the review, and the role of the funders or sponsors in the review.	Competing interests and support
Competing interests	26	Declare any competing interests of review authors.	Competing interests and support
Availability of data, code and other materials	27	Report which of the following are publicly available and where they can be found: template data collection forms; data extracted from included studies; data used for all analyses; analytic code; any other materials used in the review.	Availability of data, code and other materials
